# The Influence and Power of Association

**Published:** 1898-04

**Authors:** J. Taft

**Affiliations:** Cincinnati, Ohio


THE DENTAL REGISTER.

VOL. LII.] APRIL, 1898. [No. 4.

### COMMUNICATIONS.

### The Influence and Power of Association.

BY J. TAFT, D.D.S., CINCINNATI, OHIO.

Read before the Odontographic Society, of Chicago, February 21, 1898.

The power and influence of associated effort is recognized in all the callings and occupations of human industry and thought. The expression, "In union there is strength," finds a verification in the multiplicity of organizations which embrace a large share of the peoples of the civilized world.

People associate themselves for the accomplishment of that which could not be done by them in their individual capacity.

The world is moved by association. While it is true that some things have been accomplished by individual effort, it is equally true that the great movements of the world have been carried on by a union of forces, even in the most simple callings of life.

People associate themselves for the accomplishment of their proposed objects. This is true of those in professions, as well as in other callings and occupations.

Religious as well as secular workers and thinkers throughout the world have their organizations.

Physicians of all schools and specialties have brought this means of improvement and usefulness to a very high state of activity.

Dentists, not less than any others, have brought into operation, tested and proved the benefits and value of co-operation.

Dental societies began to be formed about fifty-eight years ago. The first step in this direction was the establishment of the pioneer organization, the American Society of Dental Surgeons. Though two attempts prior to this were made, but they were not successful. The American Association of Dental Surgeons was the first of any special importance.

This organization had a varied career of about sixteen years, when it disbanded. Though it was short-lived, it proved that organized effort might be utilized as an instrumentality of great good, and as an agent to promote the growth and prosperity of the profession. It demonstrated some valuable points to be attained by association, and as clearly indicated some points of danger, which should ever after be avoided. In both these respects it accomplished valuable service.

So promptly and generally was the value of associated work recognized that other organizations were, from time to time, established.

The Virginia Association of Dental Surgeons was formed December 12, 1842; the Mississippi Valley Association of Dental Surgeons, August 13, 1844; the Pennsylvania Association of Dental Surgeons, December 14, 1845; the Society of Dental Surgeons of the State of New York, November 17, 1847; the American Dental Convention, August 2, 1855, and the American Dental Association in 1859.

From that time onward State Associations have been organized, until every State and Territory in the Union, with possibly one or two exceptions, has its State Dental Society; and each city of a few thousand inhabitants or more can boast of its Dental Society, and some of the larger cities have two or three organizations.

So great is the importance of association work now regarded that no one of any reasonable professional ambition can afford to stand aloof from or refuse participation in it.

Association work is being utilized by dentists in a higher degree than in any other profession.

There are in this country three organizations of the National character, viz:

(1) The National Dental Association.

(2) The National Association of Dental Faculties.

(3) The National Association of Dental Examining Boards.

The special work of these need not be specified here. In addition to these, there are three district associations. These are so distributed as to embrace the entire United States, and are directly auxiliary to the National Society. Then comes the State Societies, as above specified, and in addition to these, the societies of still more local character, namely, those of cities and larger towns, and of some country districts.

While the work of each of these organizations have some objects in common, yet each has some specific work for its object.

All alike are interested and engaged in the promulgation of the science of the profession, yet there are specific phases of work that fall to the individual bodies that it is not feasible for others to assume.

The National Society has an outlook over the whole field of the profession in this country, and should suggest, and aid so far as possible, association work throughout the whole profession. It also with propriety may, and should, suggest and advise in regard to the educational work of the profession. It should also deal with all questions of the profession of a general and National character.

The three district societies are immediately auxiliary to the National, and, in addition to scientific work, may take up and deal with more practical matters, adapting their work more particularly to the needs of that part of the country where they are located.

Every State has its special society. The work of each should embrace as much of scientific and practical matters as possible. In addition to this, it should have cognizance of and guide, so far as it may, the dental legislation of its own State; see that the laws are executed, and that they accomplish the wise purpose for which they were enacted. This is a work devolving solely upon the State Societies, and by this means maintaining a good professional standard in every portion of our country.

Every State should have an interest in the educational work of our profession, and may properly make suggestions in regard to it. A large portion of the States have one or more colleges, and the interest of these is so outreaching that it should enlist the interest and consideration of all bodies whose influence might be helpful.

Associations still more local in character than those just mentioned, namely, those of large and small cities, have their functions, in the main, in the development of the science and art of the profession. This is done by the presentation and discussion of subjects through papers and addresses, with clinical work, which may embrace all the operations and processes in practical dentistry.

Formerly it was thought to be impracticable to combine the scientific and practical or clinical work in one meeting; this, however, by the recent development, is shown to be incorrect.

It has been found, as is demonstrated on this occasion, that the study of the science of the profession and its application in practical work are not incompatible, even though presented on the same occasion.

In this respect the dental profession, in its association work, has outstripped any other profession. The medical profession does not, to any extent, attempt practical work. There is nowhere presented, outside of the dental profession, an equivalent to the practical work done in our clinics.

The first presentation of clinics in our profession was made about the year 1859, in the Indiana State Dental Society, at Indianapolis. The late Dr. Wm. H. Atkinson, of New York, and Dr. P. G. C. Hunt, of Indianapolis, were the active workers in this, one of the first public clinics. This mode of instruction in our profession is one of rapidly increasing utility. It is the means by which every new and available device and invention is brought to the attention of the profession; so that the attainments in the profession are made the common possession of all who will receive.

This work is being done more and more perfectly as time goes on. The facility and methods of communicating are constantly multiplying and increasing, so that all who desire may make available the highest and most valuable improvements, discoveries and methods.

In the past, the question has often been asked, What benefit is to accrue from attendance on Dental Societies? The statements already made will somewhat answer this question, but there are other advantages that may be named, of these the social feature is not one of the least. Before associations were much in vogue members of the profession stood apart; they were isolated, little or no fraternal communication, non-communicative, absolutely reticent in regard to their methods and modes of work. In many instances a marked hostility existed. There were, of course, a few exceptions, but fifty years ago the condition here indicated was the rule. Dentists were suspicious of each other and stood antagonistic.

By association this condition has been cleared away, and now a vestige of it scarcely anywhere exists; indeed does not at all exist, except with those outside of association influence.

If nothing more than this had been gained, it is worth far more than all our association work has cost; it has made friends of those who were unfriendly; it has made co-workers of those who were widely separated and in a more or less degree hostile; but more than this has been accomplished, it has been a medium of communicating the best thought and the best knowledge and the best work in our profession to those who were needy and bringing them up to a higher standard of appreciation and attainment in the work, and thereby securing to those who were suffering from disease a better and more efficient service.

A broader knowledge always promotes a higher standard of professional character. As a result of broader attainments and broader views, objectionable methods and practices are avoided and ignored.

Every one brought under the influence of healthy association desires to be ethically upon a level with his fellows, and so avoids the pernicious practices that are so common with dentists outside of association influence.

The hateful system of quackery that, barnacle-like, attaches itself to the dental profession, is all outside of dental associations.

Association is not only helpful to those who are deficient in attainments, but is beneficial as well to those who have been more favored and those whose attainments are larger. They are constantly growing under such influences, so that there is much benefit in various directions.

Another influence exercised by association is that upon the literature of our profession. Almost the entire periodical literature comes from these organizations. Very little indeed is produced by others than the membership of societies.

A record of the proceedings of these bodies finds its way into our journals, so that association exercises a wholesome influence not only over the journalistic but the textual literature of our profession.

Our literature would have been meager indeed but for the influence of associations. They have, in some sort, exercised a censorship over it.

Every one in the country engaged in journalistic work is a member of some society. All the educational work of our profession feels the influence in a very marked degree of dental association.

No dental college could afford to ignore this influence; no dental school could exist and prosper with the influence of association in opposition to it.

For the position and status enjoyed by the dentists of the country we are indebted to Dental Societies, and largely to State Societies. It is their special work to give attention in this direction.

In the increase in number of organizations and efficiency of this work, there is a prophecy of a grandly growing future for our profession, and a clear indication that it shall grow stronger and higher and more efficient in meeting the wants of an enthralled and suffering humanity.

---

A DEEP red, scarlet tongue indicates a superabundance of phosphates and soda. Administer dilute nitro-muriatic acid to bring about an equilibrium of the salts of the body.—*Med. Summary.*

